# Colchicine therapy for deep vein thrombosis in a patient with vascular-type Behçet disease: A case report

**DOI:** 10.1097/MD.0000000000019814

**Published:** 2020-04-17

**Authors:** Daishi Nonaka, Hiroyuki Takase, Masashi Machii, Kazuto Ohno

**Affiliations:** Department of Internal Medicine, Enshu Hospital, JA Shizuoka Kohseiren, 1-1-1 Chuo, Naka-ku, Hamamatsu, Shizuoka, Japan.

**Keywords:** Behçet disease, colchicine, deep vein thrombosis

## Abstract

**Rationale:**

Behçet Disease (BD) is a chronic inflammatory vasculitis with thrombogenicity and multisystem involvement. Deep vein thrombosis (DVT) in the lower extremities is the most frequent manifestation of vascular involvement in BD. The causes of thrombosis vary widely and include congenital predisposition and acquired factors, but of all the thrombosis, the cause is rarely BD. Furthermore, there are few reports of treatment for thrombosis in BD.

**Patient concerns:**

We herein describe the case of an Asian male patient aged 40 years, admitted to our hospital for left leg pain, edema, and swelling.

**Diagnoses:**

We confirmed the DVT and pulmonary artery thrombosis (PAT) by contrast computed tomography angiogram. At the same time, the patient developed oral ulcerations and skin lesions consistent with BD.

**Interventions:**

The patient was initially treated with anticoagulants. However, because the improvement of DVT was inadequate, we added colchicine in anticipation of anti-inflammatory effects. After that, anticoagulation was discontinued, and only colchicine was continuously prescribed.

**Outcomes:**

We observed an almost complete resolution of DVT and PAT with no recurrence of thrombosis for 6 months after discharge.

**Lessons:**

This case shows us that we should consider BD as a differential diagnosis of DVT and that colchicine therapy is effective for inflammation-induced thrombosis in BD.

## 1 Introduction

Behçet Disease (BD) is a rare, chronic, relapsing vasculitis with a broad range of organ involvement and is classified under auto-inflammatory disorders. The exact etiopathogenesis of the disease is still unclear, although genetic predisposition, environmental factors, and immunologic abnormalities have been considered.^[1]^ It is classically characterized by recurrent oral aphthae (the main and the most recurrent symptom), genital ulcerations, variable skin lesions, uveitis, and peripheral arthritis. BD may involve vascular manifestations as well as neurological and intestinal manifestations. Typical vascular diseases are deep vein thrombosis (DVT) and superficial phlebitis.

DVT is thought to be caused by inherited or acquired risk factors or their subsequent interactions. Risk factors of DVT include age, obesity, surgery, trauma, systemic infection, sepsis, pregnancy, malignant disease, hormone replacement therapy, and oral contraceptives. Systemic inflammation, such as BD, may be the cause of DVT, and many of the above risk factors are involved in thrombosis formation through inflammatory mediators.^[2]^ In addition to endothelial injury, several mechanisms by which inflammation forms a thrombus are being currently studied.^[3]^

Regarding treatment of inflammation-related thrombus in BD, there is no consensus. Although there are some recommendations for anticoagulation therapy, most physicians believe that immunosuppressants are the key to successful treatment. Colchicine has been used in the treatment of BD, especially for mucocutaneous lesions,^[4]^ whereas few reports have shown its effect on vascular lesions.

We present the favorable response to colchicine treatment added to anticoagulant therapy in a BD patient complicated with DVT and pulmonary artery thrombosis (PAT).

## 2 Case presentation

A 40-year-old Asian male patient was referred to our hospital by his primary doctor due to warmth, pain, edema, and swelling in the left leg. He had experienced similar symptoms intermittently over the last 2 years, which were initially managed with non-steroidal anti-inflammatory drugs. (NSAIDs) The symptoms were accompanied by a rash, which was seen not only in the lower extremity but also around the neck and eyelids, with repeated appearances and disappearances. The patient also complained of recurrent oral ulcers. He was also sometimes aware of atypical chest pain during the 2-year period. He was suspected of having venous thrombosis by venous ultrasound at a dermatology clinic 2 months before the referral, but no evidence of venous thrombosis was found and no further treatment out with NSAIDs was required. His medical history was a surgery for the rupture of median nerve 9 years ago. He had a continuous smoking habit of 20 cigarettes a day for 20 years and had taken a small amount of alcohol every weekend. His family history was unremarkable. He had worked in the transportation industry for 20 years. His medication profile for the pain and edema of the left leg included celecoxib 200 mg twice a day and azosemide 30 mg once a day. Examination findings upon admission were as follows; his height and weight were 1.72 m and 66.0 kg, respectively (body mass index [BMI], 22.3 kg/m^2^), swelling and tenderness on his left leg and the left thigh circumference was greater than the right (42.0 vs 38.0 cm), blood pressure was 139/87 mmHg, pulse 97 bpm (regular), and a respiratory rate of 18/minute with an oxygen saturation of 97% on room air, and body temperature was 36.6°C. Furthermore, the patient had neither anemia nor jaundice, while bulbar conjunctiva was congested. On the ophthalmologist's examination, no uveitis or fundus abnormalities suggestive of BD were found. He presented with a 2-mm aphthous ulcer on the right side of the tongue. Dermatological status showed pustules, which were localized to hair follicles on the back of the thigh and fingers, blisters of the foot, left abdominal crust formation after drainage, pigmentation of the thigh, and a painful rash with infiltrative erythema on his left lower extremity. Heart sounds were normal, and no murmurs were detected. The lung fields were clear to auscultation and the abdomen was soft with normal bowel sounds. The patient was awake, alert, and oriented. His neurological examination on admission did not find any motor or sensory deficits and the cranial nerves were normal. Orthopedic physical examination showed no local inflammatory findings including an abnormal increase in synovial fluid in his knee joint.

**Figure 1:**
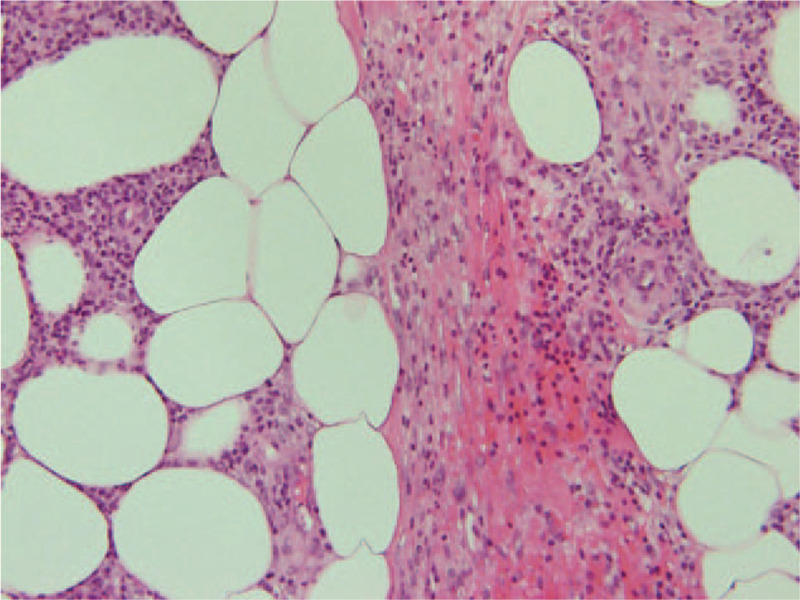
Primary skin biopsy demonstrating lobular and septal panniculitis with the infiltration of neutrophils and lymphocytes (hematoxylin-eosin, original magnification × 20).

**Figure 2:**
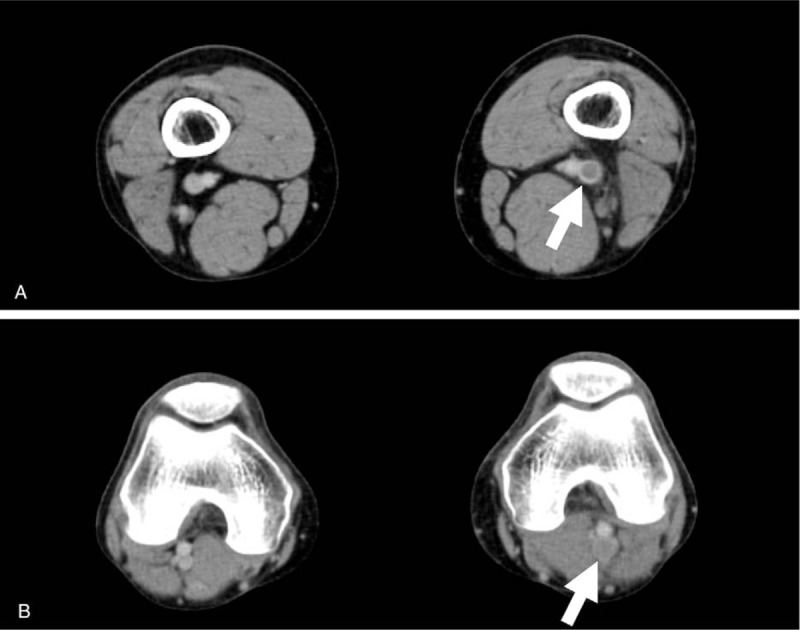
Contrast enhanced computed tomography on admission showing a thrombus from left femoral vein (A) to the left popliteal vein (B) (arrows).

**Figure 3:**
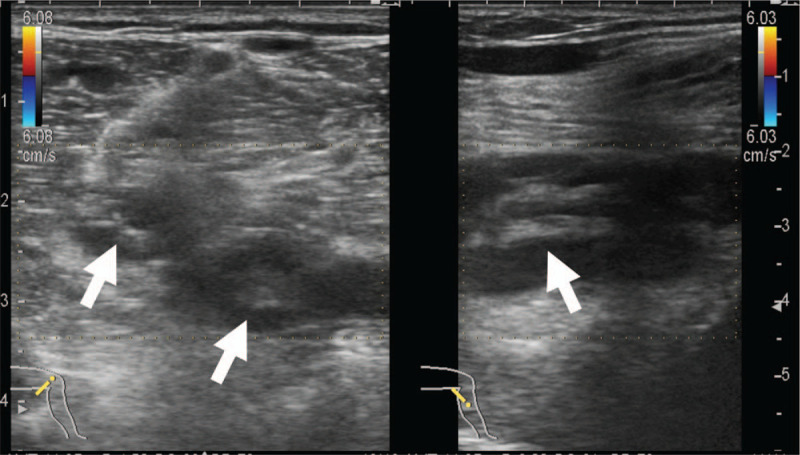
Venous ultrasonography showing an organized thrombus in the left femoral vein (arrows).

**Figure 4:**
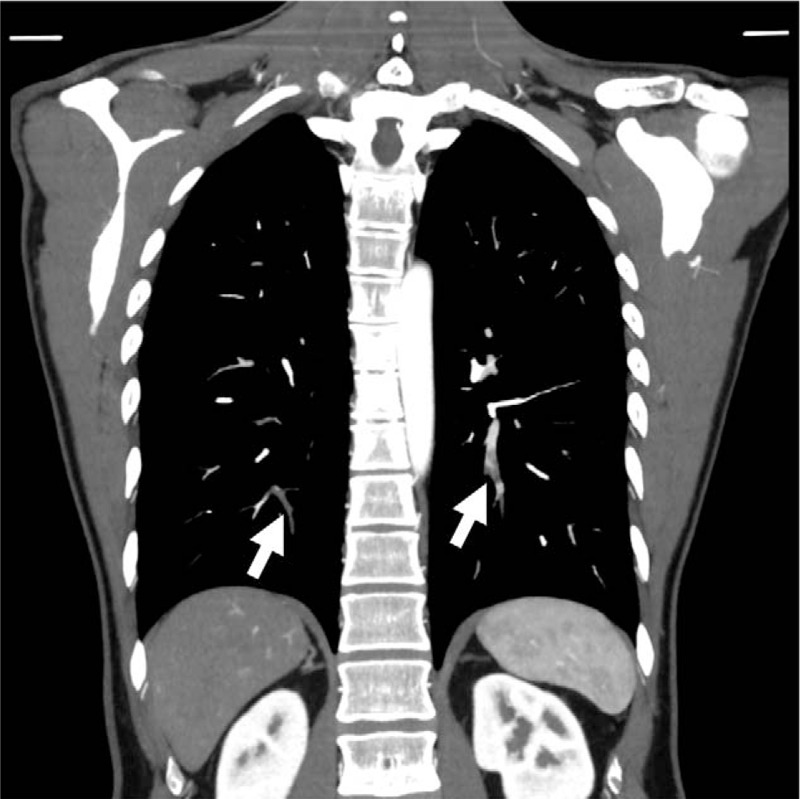
Contrast enhanced computed tomography on admission showing thrombus in the bilateral pulmonary arteries (arrows).

Laboratory tests revealed an increased white blood cells 9500/μL (normal range, 4000–9000/μL), erythrocyte sedimentation rate 18 mm/first hour (<10 mm/first hour), C-reactive protein 1.33 mg/dL (0.00–0.47 mg/dL), D-dimer, 1.7 μg/mL (<1.0 μg/mL), and fibrinogen 407 mg/dL (150–400 mg/dL). Thrombophilia investigation did not show any abnormalities, and anti-nuclear antibody, anti-DNA antibody, anti-cardiolipin IgG antibody, and myeloperoxidase and proteinase 3 anti-neutrophil cytoplasmic antibody tests were all negative. The human leukocyte antigen (HLA)-51 serologic test was also negative. The patient's laboratory data, including the above-mentioned parameters, are shown in Table 1. A bacterial culture obtained from a pustule on the back of the left thigh was negative. A skin biopsy from the left low extremity revealed lobular and septal panniculitis with variable numbers of perivascular neutrophils and lymphocytes, as well as variable numbers of necrotic adipocytes (Fig. 1). An electrocardiogram showed a normal sinus rhythm and findings on the chest radiograph were unremarkable. Transthoracic echocardiography revealed a left ventricular ejection fraction of about 60% with normal sized right-sided cardiac structures. Contrast computed tomography (CT) disclosed a thrombus from the left femoral vein to the left popliteal vein, vein wall thickening, dilatation of the entire veins, and associated inflammatory stranding of the perivascular fat (Fig. 2). Venous Doppler ultrasonography revealed that a part of the thrombus was organized, without a blood flow signal (Fig. 3). On dynamic contrast thorax CT, several clots were detected in the bilateral lower lobe branch (Fig. 4). Neither aneurysm formation nor irregular contours were seen in the pulmonary arteries. The diagnosis of PAT was also confirmed by ^99m^Tc-macroaggregated albumin scintigraphy which was presented with perfusion defects in the inferior lobe of the bilateral lung.

**Table 1:**
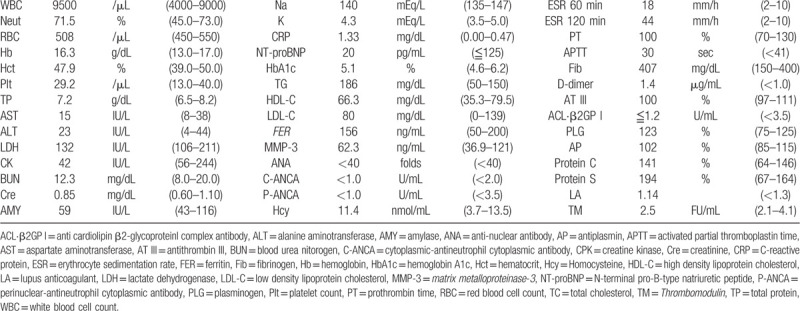
Patient's laboratory data on admission.

**Figure 5:**
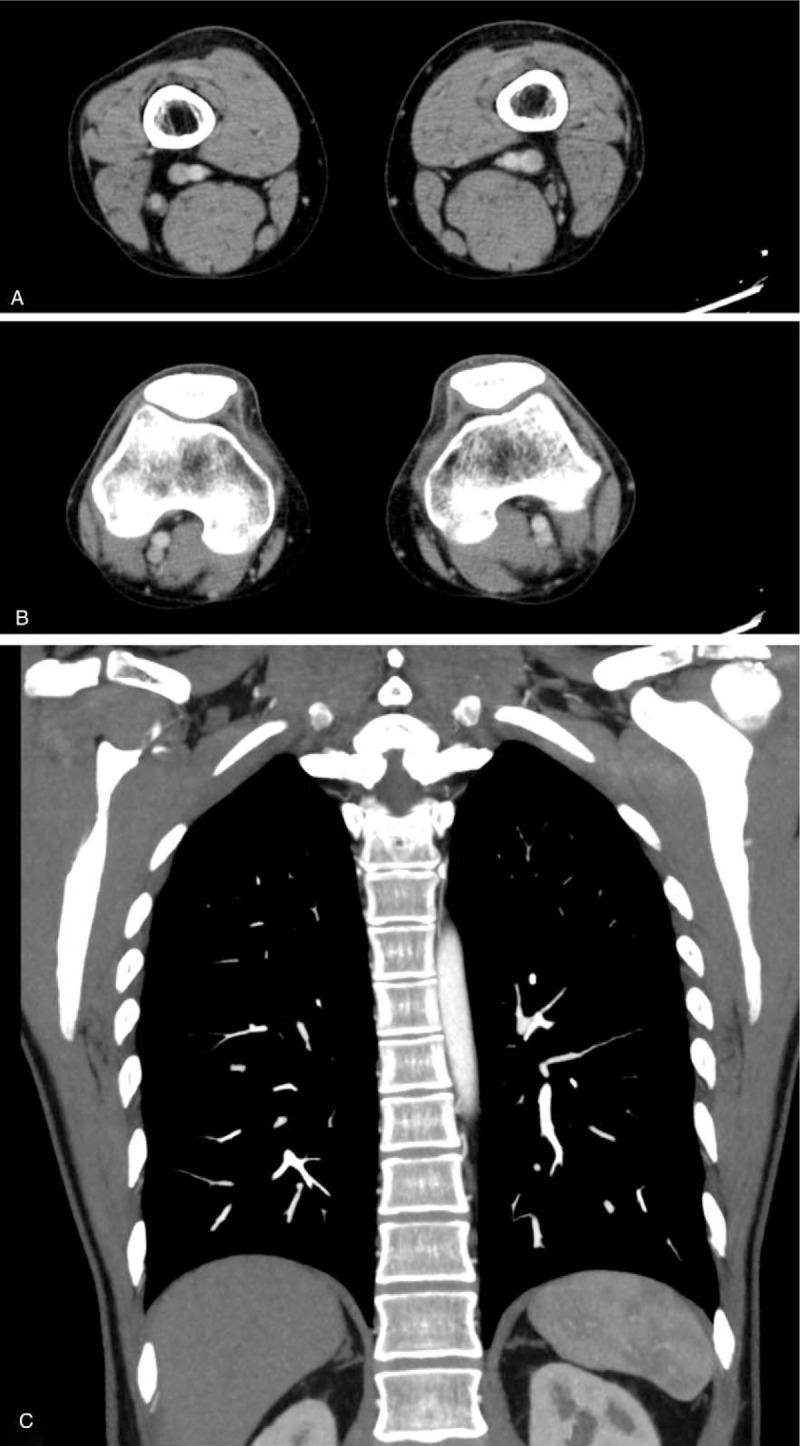
Follow up computed tomography which was performed approximately 1 month later revealed disappearance of thrombosis in the left femoral vein (A), left popliteal vein (B) and bilateral pulmonary artery (C).

On the day of admission, the patient was therapeutically anticoagulated with continuous intravenous administration of heparin sodium (18 units/kg/h), an unfractionated heparin. Initial treatment with heparin for 6 days was effective for swelling in the lower extremity, while it did not relieve the leg pain. Left thigh circumference reduced to 39.0 cm. His D-dimer level was slightly reduced to 1.4 μg/mL. Contrast CT demonstrated the reduction of clots in pulmonary arteries, but it showed no sign of improvement of the thrombus in his lower extremity. Heparin sodium therapy was switched to direct oral anticoagulant (DOAC, Edoxaban; 60 mg per day) on the seventh day of admission. At the same time, colchicine therapy for suspected BD was added to his drug regimen at a dose of 1.5 mg per day, and he was discharged on the tenth day of admission. One week after discharge, the symptoms associated oral ulcers and DVT had improved gradually. Furthermore, because cutaneous lesions, such as chronic pustules and blisters, had greatly diminished, colchicine was reduced to 1.0 mg per day in consideration of a side effect of diarrhea. Approximately 1 month after treatment with edoxaban and colchicine, the follow-up contrast CT confirmed the complete resolution of thrombus in the pulmonary arteries and almost disappearance, except for residual microthrombi, in his left popliteal vein (Fig. 5). During this period, significant improvement of the laboratory parameters of this patient was obtained as follows: white blood cells 6200/μL, erythrocyte sedimentation rate 2 mm/first hour, C-reactive protein 0.10 mg/dL, D-dimer, <0.1 μg/mL and fibrinogen 186 mg/dL. Three months after the start of oral medication, edoxaban treatment was discontinued, and only colchicine was subsequently prescribed as maintenance therapy. Colchicine, which was continued at 1.0 mg per day until that time, was to be gradually increased to 1.5 mg per day due to the instability of skin findings such as new several pustules on the right leg and the back of the right hand and new red papules on the right popliteal fossa. Infiltrative erythema was also newly identified in the right forearm, which was suggestive of superficial thrombophlebitis. Clinical and laboratory examinations revealed no signs of recurrence of DVT during the 6-month follow-up after discharge.

## 3 Discussion

We herein described a rare case, in which the cause of DVT and PAT was suspected to be BD. This patient initially presented with DVT and PAT and was treated with anticoagulants. After that, as he was diagnosed with BD due to other organ manifestations, such as oral ulcers and skin lesions; colchicine was added to DOAC for the treatment of DVT. From the course of this case, it was considered that colchicine was more effective than edoxaban, especially for DVT of the lower extremity. This case indicates to us the importance of considering BD in the differential diagnosis when encountering idiopathic DVT, particularly if dermatological findings, including recurrent aphthous ulcers or skin lesions, are present. The fact that the age of this patient with DVT of unknown cause was within the favorable onset age of BD, 20s to 40s, also helped us to diagnose the disease.

Also known as the “silk load disease,” BD cases mainly occur in the Mediterranean littoral region to East Asia. Following Turkey, Japan as well as Korea and China are reported to have the second highest prevalence of BD.^[5]^ The prevalence of vascular involvement in patients with BD varies by country and region. It in Japan is as small as 7.7%,^[6]^ but it is important as vascular involvement is the most common cause of severe morbidity and increased mortality in patients with BD.^[7]^ BD is a systemic vasculitis caused by an inflammatory response in the vessels. Vasculitis is generally characterized by differences in the size of blood vessels that cause inflammation; for example, in Takayasu's disease only large vessels are affected, while BD can affect almost every vascularized area of the body, because both arteries and veins of all sizes are involved. BD usually affects veins rather than arteries. Ishibashi reported that vascular lesions in Turkish BD patients included superficial thrombophlebitis (53%), DVT (30%), and arterial lesions (4%).^[8]^ In particular, the most common type of DVT in patients with BD is thought to be localized to the lower extremities. Thrombus of this type can cause lower extremity pain, erythema with induration, edema, hyperpigmentation, intermittent claudication, and ulceration. In fact, the thrombosis observed in this case was also localized from the femoral vein to the popliteal vein. In patients with BD, other locations of thrombus have been previously reported to be inferior vena cava as well as the renal vein and cerebral vein sinus.^[9–11]^ On the other hand, for arterial lesions in BD, the inflammatory response of arterial vessels results in the formation of aneurysm, thrombus, stenosis, and ulceration. In the current case, aneurysms of the aorta and pulmonary arteries were ruled out by contrast CT imaging. Because, in later years, the involvement of the arterial system is more frequent than that of the venous system, regular observation with appropriate modalities of intervention are necessary in the future. PAT, which was also confirmed in this case, has generally been considered to be associated with DVT in the lower extremities. However, for BD patients, venous thrombi in lower extremities are a rare cause of pulmonary artery thromboembolism because of strong adhesion to the inflamed walls. In contrast, it is considered that pulmonary artery vasculitis could result in intima injury and form a pulmonary thrombus.^[12]^ In our case, because the effect of anticoagulant therapy was limited, it was difficult to distinguish in situ pulmonary thrombus from pulmonary artery thromboembolism. Furthermore, cardiac involvement, which is a life-threatening complication, is sometimes observed in BD. The type of involvement includes intracardiac thrombosis, myocardial infarction, myocardial aneurysm, myocarditis, pericarditis, coronary arteritis, and valvular diseases. Nishida et al showed endocardial calcification in a male patient with BD in 2003.^[13]^ The patient discussed in this case showed no cardiac involvement by transthoracic echocardiography or chest CT images.

Although it has not yet been elucidated why patients with BD exhibit a thrombotic tendency, it is believed that inflammation-induced endothelial cell damage seems to be the main mechanism underlying vascular thrombosis. As previously reported, viral, bacterial, genetic, environmental, toxic, and immune triggers are considered to be involved in the mechanism of BD in complex manner.^[14]^ In physical conditions, the vascular endothelium maintains a balance between pro-coagulant and fibrinolytic status. Endothelial cell injury leads to an activation of coagulation cascade by exposing subendothelial collagen, releasing pro-coagulant agents, and suppressing fibrinolytic system.^[3]^ Plasma von Willebrand factor, which functions to bind platelets to exposed collagen in areas of damaged endothelium to promote normal hemostasis, was increased in active BD patients.^[15]^ The same authors also demonstrated that the level of plasminogen activator inhibitor leading to decreased tissue plasminogen activator was significantly higher in BD group than in non-BD group.^[15]^ Because vascular endothelial injury itself is a common event in all vasculitis diseases, including BD, it alone does not explain the increased prevalence of thrombosis in BD compared to other vasculitis diseases. It is speculated that there is some other possible mechanism in the pathogenesis of increased thrombogenicity in BD. For thrombophilic factors, a case report revealed the deficiency of protein S in the BD family.^[16]^ In the present case, no significant deficiency was observed in proteins S and C and antithrombin III. Large platelets have more potent thrombogenicity than small platelets.^[17]^ Mean platelet volume was significantly higher in patients with BD than healthy controls, and it was an independent predictor for vascular thrombosis in BD.^[18]^ Microparticles expressing tissue factor play a critical role in a variety of diseases such as cancer-associate thrombosis.^[19]^ Circulating tissue factor-positive microparticles was significantly increased in BD patients compared to normal controls.^[20]^ Furthermore, neutrophil activity has been reported to enhance the activity of reactive oxygen and affect thrombus formation in BD patients,^[21]^ and its aggregation is also observed in the histopathology of this case.

From the viewpoint that the main pathophysiology of vascular thrombosis in BD is inflammation rather than coagulation abnormality, treatment is based on the use of immunosuppressive drugs rather than anticoagulants.^[22]^ The primary objective in the treatment of BD patients is to prevent irreversible damage that can affect quality of life. In 2008 recommendations of the European League Against Rheumatism for management of BD, anticoagulant therapy received the weakest strength of recommendation based on the lowest level of evidence.^[23]^ The reason is that a single anticoagulant therapy may increase risk of fatal bleeding and hemoptysis unless detailed vascular modalities have excluded the presence of arterial aneurysm.^[12]^ Instead, colchicine, immunosuppressive agents such as azathioprine and cyclophosphamide, and monoclonal antibodies against tumor necrosis factor-a (TNF-a) are recommended.^[24]^ In this case, after confirming that there was no apparent aneurysms of the aorta or pulmonary artery, treatment with anticoagulation was started for thrombosis, but when vascular-type BD was strongly suspected, colchicine was started in combination with the anticoagulant, and 3 months after initiation of the treatment, we decided to use colchicine alone. Since there was no recurrence of thrombosis in the lower extremity and pulmonary artery up to 3 months after stopping DOAC, the anti-inflammatory action by colchicine was considered to be effective in the suppression of thrombus formation in BD. Colchicine, which is a simple, inexpensive, and nonsteroidal anti-inflammatory medicine, has been used traditionally for gout and has shown promise as a treatment for acute and recurrent pericarditis. At low concentrations, colchicine inhibits the formation of microtubules, while at higher concentrations it promotes their depolymerization.^[25]^ Colchicine impairs adhesion of neutrophils to the vascular endothelium, diminishes endothelial selectin family-dependent adhesiveness,^[26]^ and blocks the secretion of pro-inflammatory cytokines such as IL-1β.^[27]^ As a result, it leads to a reduction of inflamed area and a prevention for exacerbation of disease. Krumb et al showed that treatment with colchicine improved symptoms associated with cerebral venous sinus thrombosis in BD patients.^[11]^ In addition, 1 report demonstrated that colchicine therapy for 6 months reduced mean platelet volume in BD patients.^[28]^ There are randomized double-blind trials of BD that have shown an effect on colchicine,^[29,30]^ but participants in these studies have no major organ involvement such as vascular disease. Regarding immunosuppressants, corticosteroids, azathioprine, cyclosporine A, and cyclophosphamide are recommended in the management of DVT in BD. In the future, if recurrence of thrombosis, deterioration of skin involvement, and new appearance of multiple organ lesions are observed despite continuing the treatment with colchicine in this patient, change or the addition of immunosuppressive agents should be considered with careful attention to the complications such as nephrotoxicity and infection. In more resistant cases, treatment with (TNF-a) inhibitor, such as infliximab, is thought to be required.

Without any specific imaging feature or laboratory findings, the diagnosis or classification of BD depends on appropriate clinical symptoms and signs. In other words, some patients may have a prolonged journey from the first presentation until diagnosis of BD. In Japan, diagnostic criteria published by the Ministry of Health, Labor and Welfare is currently in use and it has been last updated in 2010. In the diagnostic criteria, the presence or absence and the combination of 4 major and 5 minor symptoms are classified into complete, incomplete, or suspected types. Because our patient had only 2 major features (recurrent oral ulcer and skin lesions) and one minor feature (vascular lesion), he was classified as both suspected-type and vascular-type BD. Although Japanese diagnostic criteria focus on ocular lesions, as with current case, patients with vascular BD are less likely to have ocular lesions.^[8]^ HLA-B51 and -A26 are known to be strongly associated to BD, but these are not included in the diagnostic criteria. Three factors, abnormalities of blood flow, blood constituents, and endothelial injury at vessel wall, proposed by Dr Rudolph Virchow are generally involved in thrombus formation and propagation.^[31]^ The differential diagnosis of DVT and PAT of unknown origin should be considered based on Virchow's triad. First, no inherent or acquired factors that may lead to venous stasis were confirmed in current case. Second, representative diseases such as underlying neoplasm and congenital factor defects that can cause hypercoagulability are also excluded by blood and CT examinations. Finally, given his age of onset and recurrence of oral ulcers, the patient's thrombosis is suspected to be due to the inflammatory response of vessel walls in BD, and the subsequent evaluation of the comorbid cutaneous lesions and the reactivity to colchicine therapy confirmed this diagnosis.

In conclusion, it should be recognized that BD could be the cause of systemic thrombosis such as DVT and PAT. Inflammation is strongly involved in the pathophysiology of thrombus formation, so colchicine may be more effective than anticoagulant therapy especially in the treatment of thrombosis in BD, and it is worth trying before immunosuppressive drugs from the perspective of few complications.

## Acknowledgments

We would like to thank Editage (www.editage.com) for English language editing.

## Author contributions

DN was involved in the making of manuscript and illustrations. HT served as advisor and editor for this case report. All authors read and approved the final manuscript.

**Conceptualization:** Daishi Nonaka.

**Investigation:** Daishi Nonaka.

**Supervision:** Hiroyuki Takase.

**Writing – original draft:** Daishi Nonaka.

**Writing – review & editing:** Daishi Nonaka.
